# Morphologic CT and MRI features of primary parotid squamous cell carcinoma and its predictive factors for differential diagnosis with mucoepidermoid carcinoma

**DOI:** 10.1186/s13244-022-01256-x

**Published:** 2022-07-15

**Authors:** Xiaohua Ban, Huijun Hu, Yue Li, Lingjie Yang, Yu Wang, Rong Zhang, Chuanmiao Xie, Cuiping Zhou, Xiaohui Duan

**Affiliations:** 1 Department of Radiology, State Key Laboratory of Oncology in South China, Collaborative Innovation Center for Cancer Medicine, Sun Yat-Sen University Cancer Center, 651 Dongfeng Road East, Guangzhou, Guangdong 510060 People’s Republic of China; 2 Department of Radiology, Guangdong Provincial Key Laboratory of Malignant Tumor Epigenetics and Gene Regulation, Sun Yat-Sen Memorial Hospital, Sun Yat-Sen University, No. 107 Yanjiang Road West, Guangzhou, Guangdong 510120 People’s Republic of China; 3 Department of Radiology, The University of Hong Kong-Shenzhen Hospital, No.1, Haiyuan Road Futian District, Shenzhen, 518000 People’s Republic of China

**Keywords:** Squamous cell carcinomas, Primary, Parotid gland, Imaging, Predictive, Diagnosis

## Abstract

**Background:**

Primary parotid squamous cell carcinoma (SCC) is a rare entity with a poor prognosis. Pathologically, the diagnosis of it requires the exclusion of parotid mucoepidermoid carcinoma (MEC). Currently, the imaging features of primary parotid SCC and the predictive indicators for differential diagnosis of the two entities have not been well reported. Our purpose was to identify the imaging characteristics of primary parotid SCC and to determine the predictive factors for its’ differential diagnosis.

**Results:**

Thirty-one participants with primary parotid SCC and 59 with primary parotid MEC were enrolled. Clinical, CT and MRI features were reviewed and compared by univariate analysis. Then, multinomial logistic regression was used to determine the predictors to distinguish parotid SCC from MEC. Most primary parotid SCCs exhibited irregular shape, ill-defined margin, incomplete or no capsule, heterogeneous and marked or moderate enhancement, necrosis, local tumor invasiveness (LTI). Age, maximal dimension, shape, degree of enhancement, gradual enhancement, necrosis, and LTI were different between the primary parotid SCCs and MECs in univariate analysis (*p* < 0.05). While in multinomial logistic regression analysis, only age and necrosis were the independent predictors for distinguishing parotid SCC from MEC, and this model exhibited an area under curve of 0.914 in ROC curve analysis.

**Conclusions:**

Primary parotid SCC has some distinct imaging features including the large tumor size, irregular shape, ill-defined margin, and particularly the marked central necrosis. Patients with age ≥ 51.5 years and necrosis on the image of the primary tumor in the parotid gland could be more likely to be SCCs than MECs.

## Key points

- Primary parotid SCC has some distinct imaging features such as the large tumor size, irregular shape, ill-defined margin, and obvious central necrosis.
- Age ≥ 51.5 years and necrosis on image are predictors for discriminating parotid SCC from MEC.
- The obtained logistic regression model is a good predictor for differential diagnosis of SCC from MEC.

## Background

Primary parotid squamous cell carcinoma (SCC) is a rare entity with an incidence varying from 0.1 to 10% in salivary malignancies [1–3]. Primary parotid SCC usually conveys a poor prognosis even with surgery and postoperative radiotherapy [1–3]. At present, the pathological characteristics, therapy and prognosis of primary parotid SCCs have been well described [1–3]. However, to date, only one literature has reported the MRI findings of primary parotid SCCs in a small sample size of just 7 patients [4]. Therefore, the imaging characteristics of primary parotid SCCs need be comprehensively described in a larger series.

In addition, it has been recommended that the diagnosis of primary parotid SCC must exclude high-grade mucoepidermoid carcinoma (MEC) or metastatic parotid SCC firstly [5, 6]. For metastatic parotid SCC, a comprehensive review of the medical history and careful examination are essential to distinguish the primary from metastatic SCC of parotid gland, because of the similar histologic features and immunoprofiles between them [7]. For MEC of parotid gland, as one of the most common parotid malignancies, it is especially important to distinguish it from primary SCC because of its much better prognosis than the latter [8, 9]. Currently, the imaging features of primary MEC in the parotid gland have been well described [10–13]. Nonetheless, there is no literature about the predictive imaging indicators for differential diagnosis of primary parotid SCC from MEC, to our best knowledge.

In the present study, we retrospectively analyzed the imaging features of primary parotid SCC and compared the imaging features between primary parotid SCC and MEC. The purpose of this work is to determine the imaging features of primary parotid SCC, with an emphasis to determine the predictive factors for differential diagnosis of primary parotid SCC from MEC.

## Materials and methods

### Patients

Thirty-one participants with pathologically proved primary parotid SCC (by surgery in 28 participants and by biopsy in 3 participants) and 59 participants with pathologically proved primary parotid MEC (by surgery in 54 participants and by biopsy in 5 participants) were retrospectively enrolled in this study from June 2013 to March 2020. The criteria inclusions were as follows: had a pathologically proved diagnosis of parotid SCC or parotid MEC; MRI or CT examinations of the neck; no previous history of SCC in other locations during the clinical course and no evidence of SCC or MEC in other regions. The study was approved by the institutional review board of Sun Yat-Sen Memorial Hospital, and patient informed consent was exempted for this type of review.

### CT and MR imaging

Plain and contrast-enhanced CT and / or MRI examinations were carried out before initial treatment in all participants. Of the 31 participants with primary parotid SCC, 13 had CT examination and 18 had MRI examination. Of the 59 patients with MEC, 35 patients had CT and 24 had MRI examinations.

Of the 48 patients with CT examinations, 29 patients were scanned on a 64-slice spiral CT (Toshiba Aquilion 64, Toshiba Medical Systems, Japan), 8 patients on a 64-slice spiral CT (LightSpeed VCT, GE Medical Systems, USA) and 11 patients on a dual-source CT scanner (SOMATOM Force, Siemens Healthineers, Germany). The major scan parameters were as follows: tube voltage/ current = 120 kV/120–250 mA, pitch = 1–1.2, field of view = 200–240 mm, matrix = 512 × 512. A contrast-enhanced CT scan was conducted after intravenous injection of nonionic contrast material (iopamiro, Shanghai Bracco Sine Pharmaceutical corporation), with a dosage of 1.0 ml/kg at a rate of 3 ml/s. 2–5 mm thick axial and coronal multiplanar reconstructions were obtained with soft tissue kernel.

MRI examination was conducted using a 1.5 T scanner (Signa Horizon LX Highspeed, General Electric Medical Systems, USA) in 14 patients or a 3.0 T scanner (Achieva, Philips Medical Systems, Netherlands) in 28 patients using a head neck synergy coil. The imaging sequences were transverse and coronal T2WI (TR/TE = 3500/100 and 2643/90 ms, respectively), transverse and sagittal T1WI (TR/TE = 50/15 and 628/18 ms, respectively) For the contrast-enhanced scan, gadopentetate dimeglumine (Magnevist, Bayer-Schering Pharma AG, Berlin, Germany) was intravenously injected at a dose of 0.1 mmol/kg, then the images of transverse, sagittal, and coronal T1WI were acquired.

### Imaging analysis

Two experienced radiologists (C.Z. and X.B., with 14 and 11 years of experience in diagnostic imaging, respectively) blinded to the pathologic information, independently evaluated all images. The radiologic features of tumor location, maximal dimension, shape, margin, capsule, contrast enhancement pattern, degree of enhancement, gradual enhancement, hemorrhage, cyst, necrosis, local tumor invasiveness (LTI), and cervical lymphadenopathy were analyzed. Tumor location was classified as superficial part, deep part, and mixed part of parotid gland. Tumor shape was categorized into round / oval or lobulated or irregular. Margin was considered well-defined or ill-defined. Enhancement patterns were graded as heterogeneous or homogeneous. Degrees of enhancement were classified as mild, moderate and marked enhancement. Mild enhancement was defined as the degree of enhancement is ≤ adjacent muscle; moderate enhancement was > the muscle but < the mucosa on MRI or the submandibular gland on CT images; marked enhancement was similar to the enhancement of mucosa on MR imaging or the submaxillary gland on CT. Gradual enhancement was defined as a tumor having more obvious enhancement on venous phase images than on arterial phase. Intratumor necrosis and cyst were determined on enhanced CT or MRI. Necrosis was defined as a region that exhibited no enhancement (CT value less than 5 HU) with an irregular border. Cyst was defined as a well-defined and rounded region which had no enhancement, with a thin, regular and smooth wall. The degree of necrosis was categorized as absent, mild (necrosis less than half of the lesion), and severe (necrosis more than half of the lesion). LTI was defined as invasion of the subcutaneous tissue, masticator massetric space, or bone. The diagnosis cervical lymphadenopathy was derived from necrosis and size criteria [14].

### Statistical analysis

To identify the diagnosis value of clinical and radiologic parameters, the patients were classified into two groups: group 1 with primary parotid SCC, and group 2 with primary parotid MEC. Statistical analysis was conducted by using software (SPSS, version 22.0, Chicago, IL, USA). The receiver operating characteristic (ROC) curve analysis demonstrated that the optimal cutoff value of age was 51.5 years. The clinical and radiologic parameters were classified as follows: age (≥ 51.5 years or > 51.5 years), sex (male or female), tumor location (superficial part, deep part, or mixed part), shape (round / lobulated or irregular), margin (well-defined or ill-defined), capsule (complete, incomplete, or no), contrast enhancement pattern (homogeneous or heterogeneous), degree of enhancement (mild, moderate, marked), necrosis (absence, mild, or severe), and the absence or presence of gradual enhancement, hemorrhage, cyst, LTI and cervical lymphadenopathy. Comparison the frequency of above variables between the two groups was used χ^2^ test in univariate analysis. Then, multinomial logistic regression was used to determine the predictors to distinguish parotid SCC from MEC. Diagnostic power was evaluated by ROC curve analysis. Odds ratios (OR) with confidence intervals (CI) were then calculated for each risk factor. *p* < 0.05 was assigned statistically significant.

## Results

### Clinical findings

All patients with primary parotid SCC comprised 19 males and 12 females between 32 and 88 years (mean age = 61.5 years). Otherwise, patients with primary parotid MEC comprised 26 males and 33 females between 5 and 72 years (mean age = 38.0 years). Specially, 4 children with primary parotid MEC were found in our series. The mean age of the adult patients with primary parotid MEC was 40.0 years. Age was significantly different between the two groups (*p* < 0.05). The optimal cutoff value for the age was 51.5 years by ROC curve analysis. However, sex was no significant difference between the two groups.

### Imaging features of primary parotid SCC and univariate analysis

**Fig. 1:**
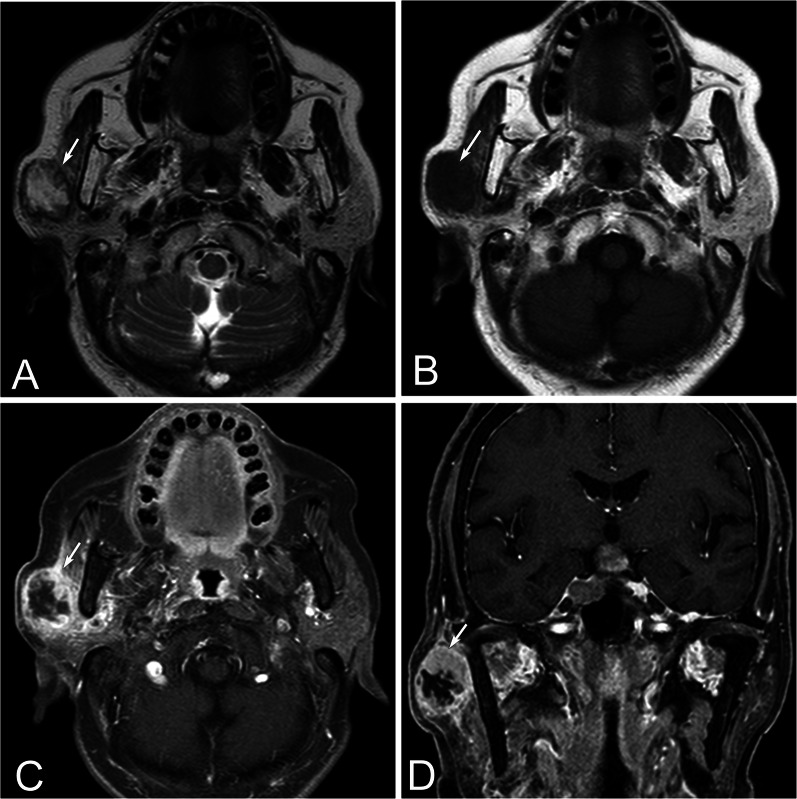
A 70 years old patient with primary parotid SCC in the right parotid gland. Axial T2WI (**A**) and T1WI (**B**) show a well-defined mass in the superficial part of the right parotid gland exhibiting a hypointense rim with central hyperintensity on T2WI, hypointense on T1WI (arrow). Contrast-enhanced axial (**C**) and coronal (**D**) T1WI show heterogeneous ring enhancement with severe central necrosis (arrow)

**Fig. 2:**
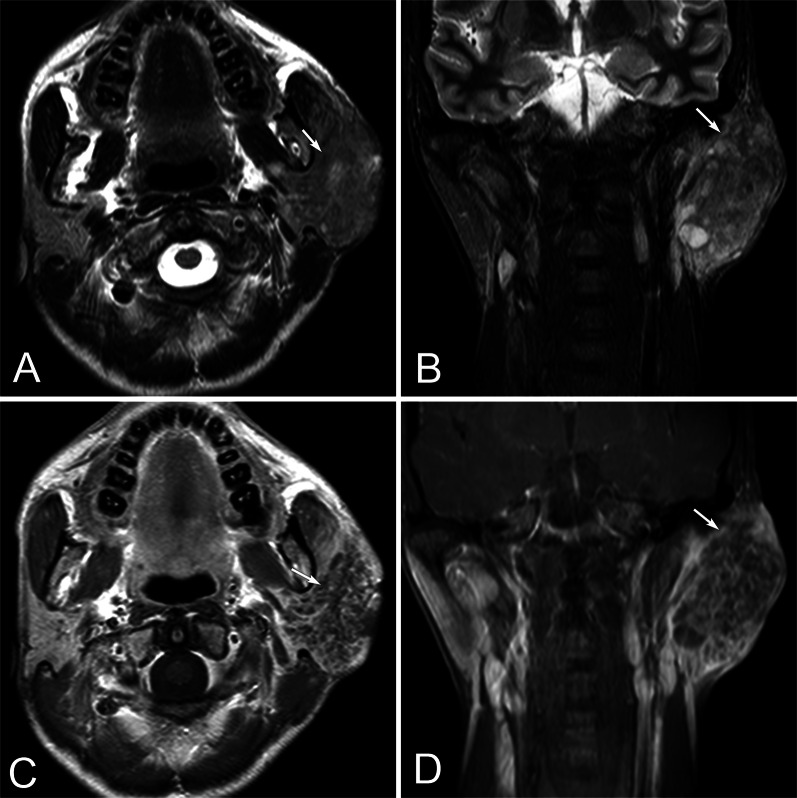
A 37 years old female with primary parotid SCC in the left parotid gland. Axial T2WI (**A**) and coronal T2WI (**B**) show an ill-defined, irregular mass in the left parotid gland. The tumor is slightly hypointense with multiple small focal areas of obvious hyperintensity on T2WI (arrow). Contrast-enhanced axial (**C**) and coronal (**D**) T1WI show obvious heterogeneous enhancement with multiple small focal areas of necrosis (arrow)

**Fig. 3:**
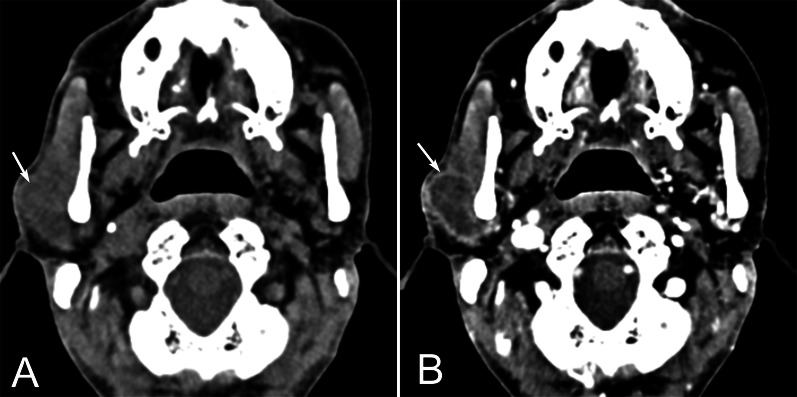
A 65 years old male with primary parotid SCC in the right parotid gland. Axial non-contrast CT (**A**) shows an ill-defined, lobulated and hypodense lesion in the superficial part of the right parotid gland (arrow). Contrast-enhanced CT (**B**) demonstrates moderate rim enhancement with severe central necrosis (arrow)

**Fig. 4:**
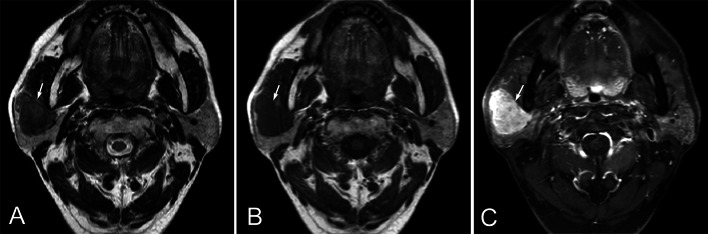
A 57 years old male with primary parotid MEC in the right parotid gland. Axial T2WI (**A**) and T1WI (**B**) show a well-defined, oval mass in the superficial part of the right parotid gland (arrow). The mass is hypointense on T2WI, and hypointense on T1WI. Contrast-enhanced axial (**C**) T1WI shows the tumor is homogeneous enhancement without necrosis (arrow)

The CT and MRI features of the two parotid gland cancers are summarized in Table 1, and the representative CT and MRI images of the two groups are shown in Figs. 1, 2, 3 and 4. For primary parotid SCC, 58% of the lesions were located in the superficial part of parotid gland, 32.2% were in the mixed part and 9.7% were in the deep part. The maximal dimension of SCCs was 1.0 to 7.6 cm with a mean of 4.12 cm. The lesion was round/ oval in 9/31 (29%) patients, lobulated in 13/31 (41.9%), and irregular in 9/31 (29%). Tumor margin was ill-defined in 20 patients (64.5%), but well-defined in 11 patients (35.5%). Most lesions (71%) were solitary, 93.5% were heterogeneous enhancement, 19.4% were gradual enhancement, 74.2% presented cyst, only 6.5% presented hemorrhage and 61.3% presented LTI. There was no capsule in 12 (38.7%) patients with SCC, incomplete capsule in 18 (58.1%) and complete capsule in only 1 (3.2%) patient. SCC had marked enhancement in 16/31 (51.6%) patients, moderate enhancement in 14/31 (45.2%) ones, and mild enhancement in 1/31 (3.1%). SCC had no necrosis in 4/31 (12.9%) patients, mild in 9/31 (29.0%) ones, severe in 18/31 (58.1%).

**Table 1:** CT and MRI characteristic and univariate analyses of primary parotid SCC and MEC

Characteristic	SCC (*n* = 31)		MEC (*n* = 59)		*p* value
	No of patients	%	No of patients	%	
Location					0.322
Superficial part	18	58.1	31	52.5	
Deep part	3	9.7	2	3.4	
Mixed	10	32.2	26	44.1	
Size (cm)*	4.12 ± 1.52		3.03 ± 1.67		0.03
Shape					0.02
Round/oval	9	29	39	66.1	
Lobulated	13	41.9	8	13.6	
Irregular	9	29	12	20.3	
Margin					0.384
Well-defined	11	35.5	27	45.8	
Ill-defined	20	64.5	32	54.2	
Capsule					0.533
Complete	1	3.2	2	3.4	
Incomplete	18	58.1	27	45.8	
No	12	38.7	30	50.8	
Enhancement pattern					0.084
Homogeneous	2	6.5	12	20.3	
Heterogeneous	29	93.5	47	79.7	
Degree enhancement					0.027
Mild	1	3.2	4	6.8	
Moderate	14	45.2	11	18.6	
Marked	16	51.6	44	74.6	
Gradual enhancement					0.020
Present	6	19.4	26	44.1	
Absent	25	80.6	33	55.9	
Hemorrhage					
Present	2	6.5	3	5.1	0.788
Absent	29	93.5	56	94.9	
Necrosis					< 0.001
Absence	4	12.9	27	45.8	
Mild	9	29.0	23	39	
Severe	18	58.1	9	15.3	
Cyst					0.065
Present	23	74.2	32	54.2	
Absent	8	25.8	27	45.8	
LTI					0.005
Present	19	61.3	18	30.5	
Absent	12	38.7	41	69.5	
Lymphadenopathy					0.108
Present	13	41.9	15	25.4	
Absent	18	58.1	44	74.6	

LTI, local tumor invasiveness

The imaging features including maximal dimension, shape, degree enhancement, gradual enhancement, necrosis and LTI were different between the two groups of parotid gland cancers (*p* < 0.05). However, the location, margin, capsule, enhancement pattern, and lymphadenopathy were not significantly different between the primary parotid SCC and MEC.

### Predictive clinical and imaging factors for differential diagnosis

**Fig. 5:**
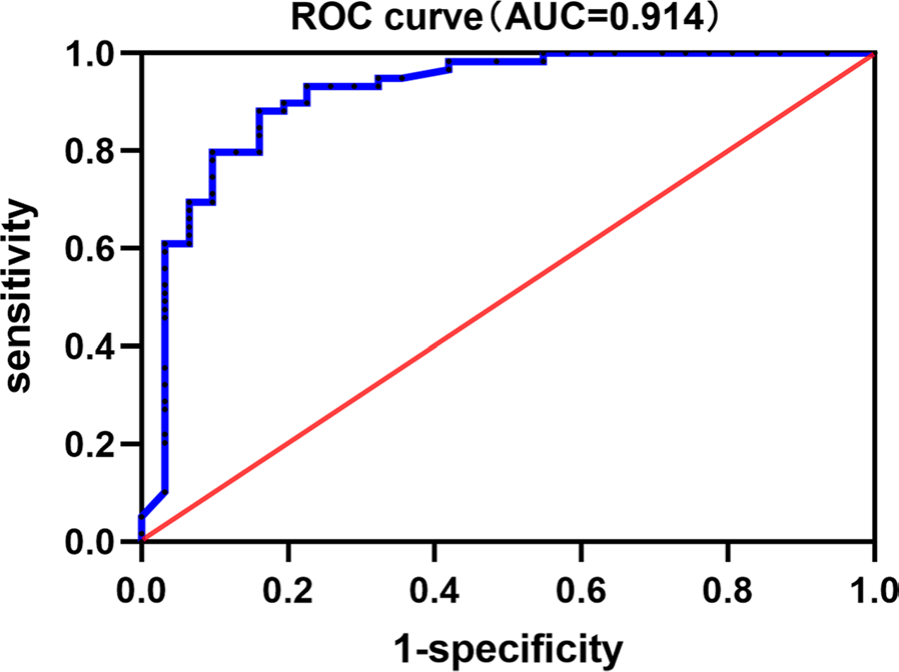
The area under the ROC curve of multivariate logistic regression model was 0.914

Only age and necrosis were independent predictors for differential diagnosis of the primary parotid SCC and MEC in multinomial logistic regression analysis (Table 2). Moreover, patients with age ≥ 51.5 years were more likely to be patients with SCC than those with MEC (OR = 18.04) (*p* < 0.001). Lesions with mild or severe necrosis were more likely to be found in patients with SCC than those with MEC (OR = 6.27, 10.22 respectively) (*p* < 0.05). The AUC of our logistic regression model in ROC curve analysis was 0.914 (95%CI: 0.845–0.984), suggesting that this model had a good ability to differentiate primary parotid SCC from MEC (Fig. 5).

**Table 2:** Multiple regression analysis of various radiologic factors

Factors	Category	*β* value	*p* value	OR (95%CI)
Size		− 0.183	0.541	0.83 (0.46, 1.50)
Shape	Oval/lobulated	0.862	0.377	2.37 (0.35, 16.05)
	Irregular	− 1.232	0.216	0.29 (0.04, 2.05)
Degree enhancement	Moderate	1.893	0.453	6.64 (0.05, 927.39)
	Marked	0.504	0.548	1.66 (0.32, 8.56)
Necrosis	Mild	1.835	0.043	6.27 (1.06, 36.95)
	Severe	2.324	0.008	10.22 (1.84, 56.79)
Gradual enhancement	Present	− 0.999	0.214	0.37 (0.76, 1.78)
LTI	Present	0.742	0.411	2.10 (0.36, 12.33)
Age	> 51.5	2.893	< 0.001	18.04 (4.01, 81.09)

LTI, local tumor invasiveness

## Discussion

In this study, our results indicated that primary parotid SCC has some distinct imaging features such as a large tumor size, an obvious invasive nature with irregular shape and ill-defined margin, and the presence of apparent central necrosis. Besides that, patients with age > 51.5 years and tumor necrosis on image are independent predictors to be primary parotid SCC. The AUC of multinomial logistic regression model in ROC curve analysis was 0.914, which suggested that it had good power to identify primary parotid SCC from MEC. Thereby, we considered that the combined use of preoperative imaging and clinical features in our model could effectively identify the high-risk patients with primary parotid SCC, accordingly more aggressive management or a wider excision can be advocated. [6, 15]

Primary parotid SCC is a rare aggressive malignancy with a worse outcome than the other common malignant tumors in parotid gland [16]. It is well established that the fine-needle biopsy, as a minimally invasive method, is useful for the diagnosis of parotid gland tumors [17]. However, it may be often difficult to correctly diagnose primary parotid SCC due to the insufficient specimens, tumorous heterogeneity and similar pathologic findings with primary parotid MEC. CT and MRI were useful for the diagnosis and differential diagnosis of primary SCC in the parotid gland. Nevertheless, literature regarding the imaging characteristics of primary parotid SCC and the differential diagnosis of it from MEC are still limited. Previously, only one study has described MRI features of primary parotid SCC in a small number of 7 cases. The results indicated that primary parotid SCC commonly shows a large tumor size, tumor necrosis, ill-defined margin, extra-parotid infiltration and low to intermediate signal intensity on T2WI [4]. In our study, we reported the highest number of patients with primary parotid SCCs so far and analyzed more imaging factors, such as tumor location, size, shape, margin, capsule, contrast enhancement pattern, degree of enhancement, gradual enhancement, hemorrhage, cyst, necrosis, LTI, and cervical lymphadenopathy. The present study yielded similar results to the previous study.[4] The tumors of primary parotid SCCs in our series tended to have more invasive growth patterns, including the large tumor size, lobulated/irregular shape, ill-defined margin, incomplete or absent tumor capsule, local tumor invasiveness and heterogeneous enhancement. Besides these above non-specific imaging features, in our opinion, central necrosis (especially the obvious necrosis) may be the relative characteristic imaging feature for the diagnosis of primary parotid SCCs. In our study, 87.1% of patients had tumors with central necrosis on CT or MRI, and more than half of patients (58.1%) had tumors with obvious central necrosis (> 50% necrosis of the tumor). This finding was similar to several previous studies, which have shown that the large cervical SCCs tended to generate central necrosis in either the primary tumors or metastatic lymph nodes. [18, 19]

To date, the predictive imaging factors for differential diagnosis of primary parotid SCC from MEC have not been reported. In the present work, we compared the clinical and imaging features between primary parotid SCC and MEC. The results showed that only age and tumor necrosis were independent predictors for differential diagnosis of primary SCC from MEC in parotid gland. Clinically, it is well known that parotid SCC was most common in males and old people (> 60 years). [2, 3, 6] In our series, 61.3% of our participants with primary parotid SCC were males with an average age of 61.5 years, similar to these reports. [2, 3, 6] Only age was an independent predictor for distinguishing the two histological groups, while the sex distribution was not different between the two malignancies. Patients with primary parotid MEC in our series had an average age of 38.0 years, which is younger than some previous reports. [10–12] This might be due to a selection bias since patients in our series concluded pediatric cases [9]. Patients with primary parotid SCC were older than that with primary parotid MEC in the present study. The optimal threshold for age was 51.5 years according to ROC curve analysis. Moreover, patients with age ≥ 51.5 years were more likely to be primary parotid SCC than those with primary parotid MEC.

In the present study, the imaging features including size, shape, degree enhancement, gradual enhancement, necrosis and LTI in lesions were found to be different between the primary parotid SCC and MEC in univariate analysis. However, in multinomial logistic regression analysis, only tumor necrosis detected on CT or MRI was an independent imaging predictor for differential diagnosis of primary SCC from MEC in parotid gland. Patients with mild or severe necrosis on CT/MRI were more likely to be patients with primary parotid SCC than those with MEC. Tumors in primary parotid SCCs tend to have central necrosis, compared with that in the primary parotid MEC. The obvious tumor necrosis of primary parotid SCCs was in line with other cervical SCCs, which were validated by microscopic pathologic features of degenerative tissue and cellular collapse [4]. The SCCs of the head and neck frequently have areas of central necrosis, surrounded by regions of low oxygen concentration (hypoxic regions). The hypoxic regions of the tumor commonly expressed hypoxia-inducible factor-1α and programmed death ligand 1, which may induce a more aggressive nature and a poorer prognosis of primary parotid SCCs than other common malignant parotid tumors [20].

There are some limitations in our study. First, this study has its retrospective nature with limited patients because primary SCCs in parotid gland are rare. The 31 patients with primary parotid SCC in our cohort were included just according to the medical records. Unavoidably, a few patients might forget to mention a previous removal of SCC of the scalp or other locations many years ago, which might result in a selection bias. Second, no advanced CT and MRI techniques such as dual-source or spectral CT, functional MRI imaging (including DWI, dynamic contrast-enhanced MRI) were used in this study. CT spectral quantitative parameters are useful for differentiating the tumors of parotid gland [21]. Additionally, DWI has high accuracy, good sensitivity and moderate specificity for the diagnosis of malignant parotid tumors [22]. Therefore, future prospective research with a larger study size or using state-of-the-art CT and MRI is demanded.

## Conclusion

Primary parotid SCC has some distinct imaging features such as a large tumor size, an obvious invasive nature with irregular shape and ill-defined margin, and particularly the marked central necrosis. Patients with age ≥ 51.5 years and necrosis on the image are independent factors for distinguishing SCCs from MECs in parotid gland. The prediction model based on the imaging and clinical factors has the potential to preoperatively detected high-risk patients of the primary parotid SCC.

## Data Availability

The datasets used and analyzed during the current study are available from the corresponding author on reasonable request.

## Abbreviations

SCC: Squamous cell carcinoma
CI: Confidence intervals
LTI: Local tumor invasiveness
MEC: Mucoepidermoid carcinoma
OR: Odds ratios
ROC: Receiver operating characteristic
